# Resilience among older adults with multimorbidity using the Connor-Davidson scale in the Canadian Longitudinal Study on Aging: health behaviour, socio-economic, and social support predictors

**DOI:** 10.1186/s12889-024-19992-8

**Published:** 2024-09-20

**Authors:** Andrew Wister, Lun Li, Jennifer Ferris, Boah Kim, Katarzyna Klasa, Igor Linkov

**Affiliations:** 1 Gerontology Research Centre & Department of Gerontology, Simon Fraser University, 2800-515 Hastings Street, Vancouver, BC V6B 5K3 Canada; 2 School of Social Work, MacEwan University, 9-510A2, 10700 104 Ave NW, Edmonton, AB T5J 4S2 Canada; 3 Gerontology Research Centre, Simon Fraser University, Vancouver, BC V6B 5K3 Canada; 4 BC Observatory for Population & Public Health, BC Centre for Disease Control, Vancouver, BC V5Z 4R4 Canada; 5 Department of Gerontology, Simon Fraser University, Vancouver, BC V6B 5K3 Canada; 6 Department of Health Management and Policy, School of Public Health, University of Michigan, Ann Arbor, USA; 7 Engineering Research and Development Center, Army Corps of Engineers, Vicksburg, USA

**Keywords:** Multimorbidity, Resilience, Aging, Health Behaviours, Connor-Davidson Scale, CLSA

## Abstract

**Objective:**

Multimorbidity is recognized as a serious health condition faced by a majority of older adults. Research investigating adaptive responses to multimorbidity, termed multimorbidity resilience, has been growing. This paper examines protective and risk factors, with a focus on health behaviours, socio-economic resources, and social support using an established measure of resilience (Connor-Davidson Resilience Scale) among older adults, focusing on older persons with two or more concurrent chronic conditions.

**Methods:**

Using Baseline (2011–2015), Follow-up One (2015–2018), and Follow-up Two (2018–2021) data from the Comprehensive Cohort of the Canadian Longitudinal Study on Aging, we tested hypotheses using 13,064 participants aged 65 years and older, who completed all waves and reported two or more of 27 chronic conditions, for the full sample of multimorbid individuals and three multimorbidity clusters: Cardiovascular/Metabolic, Musculoskeletal, and Mental Health. Associations between protective and risk factors and resilience were examined using linear regression to model the Connor-Davidson resilience scale, adjusting for illness context and social determinants of health.

**Results:**

Among all multimorbid individuals, the strongest associations with resilience were found for higher self-rated health, greater sleep satisfaction, better appetite, higher household income, more relatives and friends, being overweight (compared to normal weight), fewer housing problems, and fewer skipped meals. Weaker associations were found for non-smokers, less alcohol consumption, less pain, sedentary behaviour, being non-married (compared to married), and among Canadian born (compared to foreign). The analyses for the three multimorbidity clusters were largely replicated for the three multimorbidity clusters, but with some nuances depending on the cluster.

**Discussion:**

This research provides confirmatory evidence for several protective and risk factors affecting the ability to cope and recover from multimorbidity adversity among older adults. There are consistent patterns for the multimorbidity disease clusters, but some distinct relationships arise that are worthy of attention. The implications of the findings for modifiable health behaviours and socio-economic factors are discussed for their public health and clinical relevance.

## Introduction

Among higher-income countries, approximately two-thirds of older adults have multimorbidity (two or more concurrent chronic conditions) and these rates increase with advanced age [27, 53, 66]. Multimorbidity, often defined as the co-occurrence of two or more chronic illnesses, can have potential compounding deleterious effects that shape symptom burden, functional ability, quality of life, and result in higher health care costs [1, 26, 27, 33, 54]. Yet, research is only beginning to recognize strength-based responses among individuals (e.g., enhancing health behaviours, fostering social participation, and developing a more positive attitude) who may have been considered as not maximizing their health due to multimorbidity [57, 83]. The ability to respond positively to adversities of any kind, based on internal and external resources needed to cope with and navigate stress-inducing experiences, is termed resilience [31, 85, 95]. *Multimorbidity resilience *(MR) specifically refers to the ability to bounce back from multiple mental and/or physical chronic illness-related challenges [70, 93, 94, 103]. However, there remains a gap in research knowledge pertaining to predictors of MR, in particular, analyses of protective and risk factors, in particular modifiable health behaviours, socio-economic and social support resources or deficits [64, 71, 92]. Extending our understanding of prevention entails combining research that identifies specific predictors of multimorbidity with those that shape resilience processes such that individuals can live well with complex illness profiles. Confirmatory studies that examine predictors of resilience among older persons with multimorbidity are particularly important since modifiable predictors of coping and forms of positive adaptation to, and recovery from, multimorbidity can have significant public health implications, as well as potentially lower healthcare costs [23, 25]. This study addresses the question: what are the modifiable (behavioural lifestyle, socio-economic, and social support) protective/risk factors that are associated with resilience among persons with multimorbidity?

Examination of resilience processes of adaptation and recovery have been connected to a comprehensive number of factors embedded in socio-environmental systems [15, 28, 39, 42, 43, 73, 94, 99]. The present research is framed by the Lifecourse Model of Multimorbidity Resilience (LMMR), which views MR as a response to adversity that transitions from adverse life events, to disruption, to activation of resources embedded in the individual, social and physical environment, leading to the final process of wellness-recovery/growth [99]. One important component of this model is the role that health behaviours, socio-economic and social support resources play in fostering resilience among persons with multimorbidity, which comprise the focus of this study. This model was selected because it incorporates other common models, such as the social-ecological, social determinants, and related models, and has been used in several other studies of multimorbidity resilience [71, 103].

Social determinants of health (SDoH) are the circumstances and non-medical factors in the environment—such as where individuals are born, live, learn, work, play, worship, and age—that influence health, functioning, and quality-of-life outcomes and risks [87, 105]. These SDoHs can enhance healthy-aging and in turn affect the resilience of individuals in later life. Applied to the current study, it is well-known in gerontology that some individuals are more likely to exhibit various protective factors, such as healthy lifestyle routines, economic resources, and social support systems that may enable them to cope better than others with similar multimorbidity challenges or deficits [45, 46, 50, 62]. Protective and risk factors of resilience include a range of known of multimorbidity predictors (e.g., health behaviours, social, environmental, cultural, age, sex, gender, etc.), some of which are modifiable (health behaviours, socio-economic status, living/housing environment, assistive technology), and some of which are not (age, ethnicity, genetics) [4, 31, 53, 65]. Among studied health behaviours, smoking, physical activity, obesity, eating habits, nutrition and alcohol consumption are associated with multimorbidity [2, 5, 12, 20, 53, 65, 98]. Canizares et al. [12] demonstrated that multimorbidity risk was associated with being obese, a smoker, and engaging in a sedentary lifestyle. Other studies found associations between smoking and multimorbidity [18, 24, 52, 81], as well as poor eating habits and obesity and multimorbidity [2, 44]. While obesity is not a health behaviour per se, we consider it a marker of lifestyle factors affecting multimorbidity and resilience, such as eating habits and physical activity. The research support for associations between physical activity and multimorbidity is less clear [5, 12, 20, 30], as well as for alcohol consumption [65]. However, these behaviours, especially sedentary behaviour, may be important for resilience among persons with multimorbidity. A positive influence of sleep patterns has been found for multimorbidity recovery [23, 103], and research indicates that individuals with sleep disturbances progress to multimorbidity more rapidly [74]. Taken together, health behaviours assist in the management and adaptation to illness-related stressors, foster stronger social connections and support, and enhance well-being ([12, 36, 56].

Another primary SDoH, socio-economic status (SES), includes income, education, employment status—coupled with aspects of housing and neighbourhood contexts, factors that have been associated with multimorbidity among older adults in multiple studies [13, 32, 45, 47, 53]. SES is a critical SDoH to consider since it affects the risk of multimorbidity, frailty, and disability [21]. Additionally, housing insecurity is an indicator of socio-economic deprivation among the elderly, and this factor is estimated to grow over the next decade, particularly with the increasing affordable housing crisis in North America and Europe [7, 11, 22]. Initial research demonstrates that these SES resources or deficits can act as protective or risk factors for resilience among persons with multimorbidity [103]. Furthermore, social support, social participation and social networks have been linked to better health outcomes, including coping with multiple chronic illnesses since a supportive network can buffer stressors embedded in multimorbidity experiences [35, 46, 62, 70].

The multimorbidity literature has incorporated a number of demographic covariates that have also been been framed by a SDoH model, and include age, sex, marital status, immigration status, and urban/rural residence [12, 53, 62, 65, 70, 75, 86]. However, not all research findings have been consistent in the prediction of multimorbidity based on socio-demographic factors. Studies have shown that differences in health outcomes and multimorbidity vary according to immigration status (native-born, immigrant). However, these findings have been mixed with researchers noting both a positive health paradox among immigrants despite lower SES, possibly due to health selection effects or convergence towards native-born populations [8]. We therefore hypothesize that the above health behaviours, socio-economic status and social support will be positively (protective factors) or negatively (risk factors) associated with levels of resilience among older adults with multimorbidity.

## Methods

### Data and sample

Our study was conducted based on the Comprehensive cohort of the Canadian Longitudinal Study on Aging (CLSA). The CLSA is a national population panel study that contains a various series of information related to aging Canadians regarding their social aspects of life, and psychological, biological, and clinical health and wellbeing. Currently, three waves of CLSA data were available: Baseline data on 51,338 participants (2011 to 2015) aged 45–85, Follow-up One (FUP1) with 44,817 participants (2015 to 2018), and Follow-up Two (FUP2) with 40,305 participants (2018 to 2021), separated by approximately three years within each wave of data collection. The CLSA is comprised of two cohorts of participants, the Comprehensive cohort who were randomly selected among the population residing within 25 km (or 50 km in a lower population density area) of the 11 data collection sites across Canada, and the Tracking cohort who were randomly selected from the ten provinces by the computer-assisted interview system. Detailed information about the CLSA has been published elsewhere [38, 59, 60]. Researchers can access the de-identified data, and information on weighting through the CLSA website (www.clsa-elcv.ca).

**Fig. 1:**
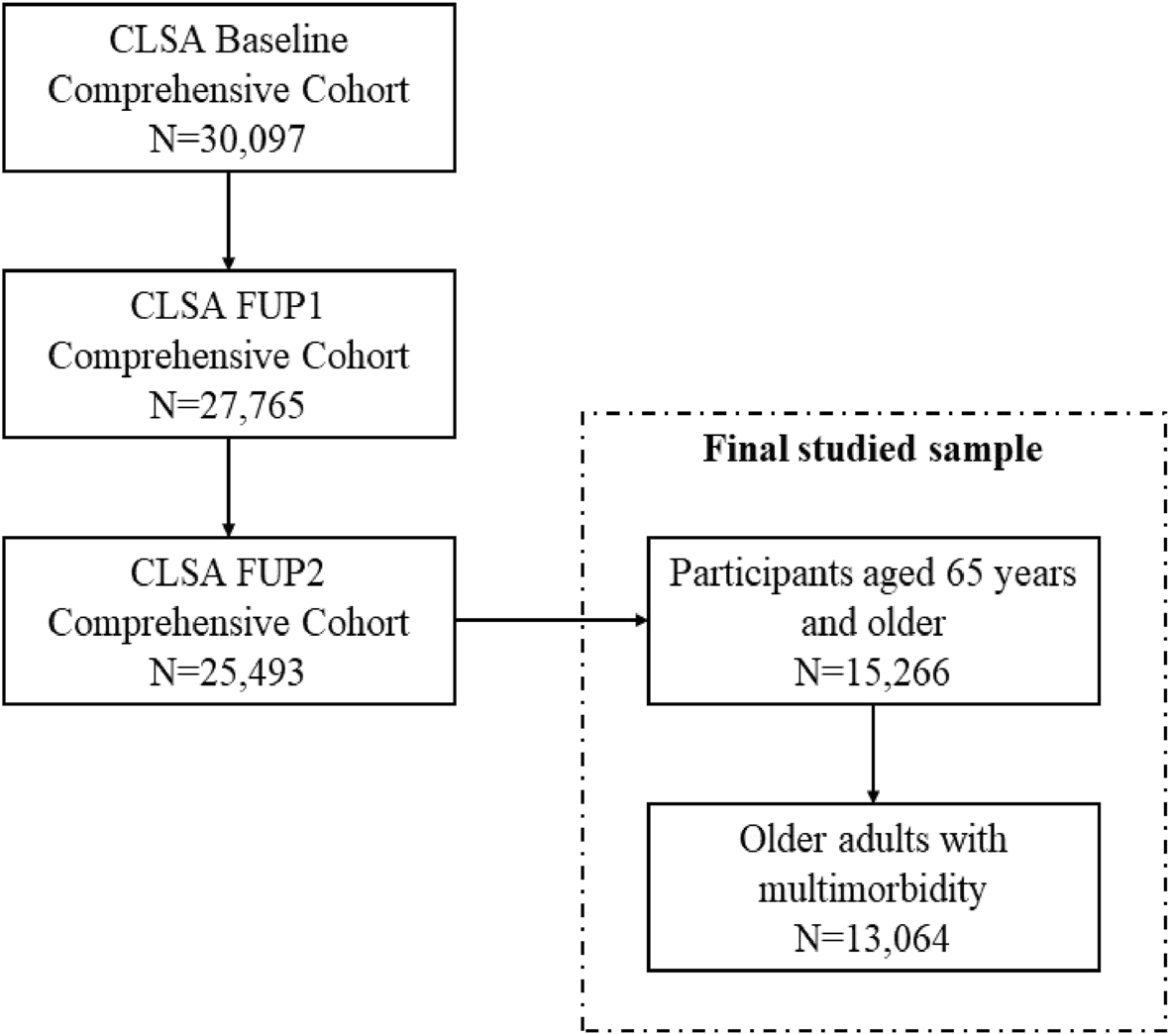
Flow Chart of CLSA Sample

This cross-sectional study was performed based on the most recent data from FUP2 for participants who completed all three waves. At the Baseline wave, there were 30,097 Comprehensive cohort participants, 27,765 further attended the FUP1 survey, and 25,493 then finished the FUP2 survey. This study focuses on older adults with multimorbidity, defined as self-reported diagnoses of two or more following 27 types of chronic conditions, including Alzheimer’s disease, back problems, bowel incontinence, cancer, cataracts, diabetes, epilepsy, glaucoma, heart attack, heart disease, high blood pressure, irritable bowel syndrome, kidney disease, Parkinson’s disease, peripheral vascular disease, lung disease, macular degeneration, multiple sclerosis, osteoarthritis, osteoporosis, migraine headaches, rheumatoid arthritis, stroke, thyroid problem, transient ischemic attack, ulcer, and urinary incontinence. We only used the Comprehensive Cohort data, given availability of the dependent measure. Thus, there are 15,266 participants aged 65 years and older at the FUP2, which leads to 13,064 older adults with multimorbidity as the target sub-sample for this study. Figure 1 provides the flow chart of CLSA participants used in this study.

We further analyzed the data based on three separate groups of participants (65 +) with two or more chronic conditions that cluster together based on priori factors. These include only highly prevalent illness types found to co-occur in the multimorbidity literature [101]. These grouping were based on review of the cluster literature and the likely co-occurrence of particular chronic illness groupings, rather than a data-driven approach used by some researchers often with inconsistent clustering identified in the data [76].

The three clusters are: 1) Cardiovascular/Metabolic cluster (heart disease, diabetes, and high blood pressure) (*n* = 3,828), 2) Musculoskeletal cluster (osteoarthritis, osteoporosis, and lower back problem) (*n* = 3,691), and 3) Mental Health cluster (mood disorder, anxiety disorder, and migraine headache) (*n*= 1,158). The three chronic illnesses within each cluster have been found to frequently co-occur [17, 29, 37, 40, 56, 68, 76], and share similar symptoms and behavioural/environmental risk profiles [6, 10, 49, 55, 61, 106]. We did not include an ‘Other’ category with all remaining illness grouping, since interpretation of this highly heterogenous group would be difficult.

### Measurement

We use Follow-up Two data for all analyses since CD-RISC 10 is only available in the most recent wave (except immigration and education, which are only available in Baseline).

#### Dependent variable

We employ the Connor-Davidson Resilience Scale 10 item scale (CD-RISC 10) [16] that measures the degree to which individuals perceive that they can overcome stress and adversity in life through a set of 10 questions, each of which is scored using a five-point scale (1–5), resulting in an interval scale with a range between 5 and 50. This measure is only available in FUP2 of the Comprehensive Cohort of the CLSA. Questions items include: 1) I am able to adapt when changes occur,2) I can deal with whatever comes my way; 3) I try to see the humorous side of things when I am faced with problems; 4) Having to cope with stress can make me stronger; 5) I tend to bounce back after illness, injury or other hardships; 6) I believe I can achieve my goals, even if there are obstacles; 7) Under pressure, I stay focused and think clearly; 8) I am not easily discouraged by failure; 9) I think of myself as a strong person when dealing with life’s challenges and difficulties; and 10) I am able to handle unpleasant or painful feelings like sadness, fear, and anger. Several of the scale items are relevant to disease/illness and the others to adversity in general. The CD-RISC_10 has excellent psychometric properties and has been used extensively in epidemiological studies, including adult and older adult populations (Scali et al. [67]; , Tourunen et al. [84]). Within the studied sample, the CD-RISC_10 has a high level of internal consistency (Cronbach’s Alpha = 0.88).

#### Independent variables

##### Socio-demographic variables

A total of six socio-demographic covariates were included: age, sex, education, household income, marital status, and immigration status. Age, sex, education and immigration status were extracted from the Baseline, and others were from FUP1. Participants’ age was measured in single years and ranged from 65 to 93. Sex was measured as “male” and “female.” The highest educational attainment was coded at four levels from “no post-secondary education”, “trade certificate or diploma or equivalent,” “bachelor’s degree,” to “university degree above bachelor’s degree,” and dichotomized into no post-secondary and post-secondary education due to small numbers in some categories. The annual household income was categorized into five groups, including “less than $20,000,” “$20,000 to $49,999,” “$50,000 to $99,999,” “$100,000 to $149,000, and “$150,000 and over.” Marital status was originally collected based on five categories, and further recoded into two groups as “not married” (single, never married, widowed, divorced, separated) and “married/common-law.” Immigration status was based on participants’ country of birth and grouped into “immigrants” and “born in Canada.”

#### Social and environmental variables

Four social and environmental covariates were included: number of friends, number of relatives, housing problems, and residential area. In the CLSA, participants were asked about the number of people they considered as close friends with whom they shared personal matters (ranging from 0 to 90), and the number of living relatives (ranging from 0 to 100). Participants who reported at least one of the seven housing-related problems (noise, leaking, condensation, electrical wiring or plumbing, heating, maintenance or repairs, and infestations) were grouped into “with housing problem(s),” and others into “no housing problem.” Residential areas were coded dichotomously as “rural area” and “urban area” based on Statistics Canada definitions.

#### Behavioural and lifestyle variables

We also incorporated six health behaviour variables, including sedentary behaviour, alcohol consumption, smoking, sleep, appetite, and skipped meals. A measure of sedentary behaviour from the Physical Activity Scale for the Elderly (PASE) [78, 89]. Participants were asked about the daily amount of time for sitting activities, ranging from “less than 1 h,” “1 h but less than 2 h,” “2 h but less than 4 h,” and “4 h or more.” Alcohol consumption is an aggregated variable based on a series of variables capturing participants’ consumption (by drinks) of beer, wine, liquor, and other types of alcohol during both weekdays and weekends. The National Institute on Alcohol Abuse and Alcoholism [51] guidelines were used to group the variable at two levels: “14 or less drinks per week” and “15 or more drinks per week,” since this cut-off reflects potentially problematic alcohol consumption. Smoking was measured based on participants’ smoking activities during the past 30 days before taking the survey. A dichotomous variable was created as “smoked” and “not smoked in the last 30 days.” Participants were asked to evaluate their sleeping quality at five levels: “very dissatisfied,” “dissatisfied,” “neutral,” “satisfied,” and “very satisfied.” Appetite was similarly self-reported and measured as “poor,” “fair,” “good,” and “very good.” An additional variable was available capturing frequency of skipped meals. This variable was recoded as: “all the time to sometimes,” and “rarely or never.” All the variables were extracted from the FUP2 in the data analysis.

#### Health context variables

Finally, three health-related variables were examined, including Body Mass Index (BMI), self-rated health and pain. BMI was categorized into four levels: “underweight” (18.49 or below), “normal” (18.5 to 24.9), “overweight” (25 to 29.9), to “obese” (30 or higher). Self-rated health was measured using a single ordinal scale categorized as “poor,” “fair,” “good,” “very good,” and “excellent.” Pain was measured based on responses to the usual intensity of pain or discomfort: “none,” “mild,” “moderate,” and “severe.” All the variables were extracted from the FUP1 in the data analysis.

### Data analytic procedure

Quantitative analytic approaches to the study of MR have examined differences in resilience among persons with and without multimorbidity, or they have focused on predictors of resilience among populations with multimorbidity only [71, 103]. To date, there are no measures of multimorbidity resilience anchored to the presence of chronic conditions (ie., items asking about resilience connected specifically to multimorbidity). In a recent scoping review of multimorbidity resilience, Seong et al. [71] identified 14 studies, of which eight were quantitative. In the eight quantitative studies, the measures used included one of the versions (10 or 25 items) of the Connor-Davidson Resilience scales [16], the short Resilience Scale [62], the Multimorbidity Resilience Index [100], the Simplifies Resilience Score [34], a life satisfaction scale [97, 101], and shorter variations of these established scales (see (Seong et al. [71])for full description). We have employed an analytic approach in which we examine modifiable predictors of resilience using the CD-RISC 10 [16] as the outcome variable and include only older persons with multimorbidity to narrow the focus to older people facing this specific adversity (multimorbidity). This approach is consistent with the conceptual definition and modeling of resilience and has been used in prior studies of MR predictors [28, 101, 103].

The Connor-Davidson resilience scales (including the CD-RISC10) are the most used measures of resilience that have been translated with approval in 90 countries and have been used in hundreds of publications (https://www.connordavidson-resiliencescale.com/about.php). The current paper produces results that can: a) be compared to other measures of resilience used in multimorbidity research; and b) compared to the use of the CD-RISC 10 in other areas of resilience adversity focusing on health behaviours and resilience outcomes.

First, descriptive analysis was conducted to show the characteristics of participants, as illustrated in Table 1. Linear regression analyses of the CD-RISC_10 score measured at FUP2 was performed using predictors measured at FUP1 (3 years prior) or Baseline (6 years prior) if an attribute such as age or sex was used. Regressions were conducted for the full sample of multimorbid participants (Table 2), followed by the three chronic health clusters (Table 3). The linear regression adjusted the socio-demographic factors, social and environmental factors, behavioural and lifestyle factors, and the health context factors. As recommended by the CLSA methods group (https://www.clsa-elcv.ca/), the trimmed weights were applied for descriptive analysis, and the analytic weights were applied for multivariate analyses (i.e., regression). All the variables contain limited missing values, except the household income variable, which contains 8.6 percent missing values (over the 5% threshold), and therefore, missing cases were replaced with “not stated.” Data analyses were conducted using SPSS version 29.

**Table 1:** Social demographic information of participants (*N* = 13,064)

Variables	Mean (SD)/percentage
**Age**	73.75 (6.73)
**Gender**	
Male	42.90
Female (ref.)	57.10
**Education level**	
No post-secondary education	44.67
Post-secondary education	55.33
**Household income**	
Less than $20,000 per year (ref.)	6.06
$20,000 to $49,999 per year	30.28
$50,000 to $99,999 per year	36.11
$100,000 to $149,000 per year	12.17
$150,000 and over per year	6.82
Not stated	8.57
**Marital status**	
Not Married	34.64
Married/Common low (ref.)	65.36
**Immigration status**	
Born in Canada	79.07
Immigrants (ref.)	20.93
**Number of friends**	5.18 (6.25)
**Number of relatives**	29.53 (25.26)
**Housing problem**	
No	79.71
Yes	20.29
**Urban/Rural status**	
Rural area	3.86
Urban area (ref.)	96.14
**BMI**	
Underweight	1.21
Normal (ref.)	28.62
Overweight	39.42
Obese	30.75
**Sedentary**	
Siting less than 1 h	0.88
1 h to less than 2 h	7.14
2 h to less than 4 h	28.50
4 h and more	63.48
**Alcohol consumption**	
14 drinks or less per week	93.02
15 drinks or more per week	6.98
**Smoking**	
Not in the last 30 days	94.71
Smoked (ref.)	5.29
**Sleep**	
Very satisfied	22.56
Satisfied	38.65
Neutral	15.37
Dissatisfied	17.04
Very dissatisfied (ref.)	6.37
**Appetite**	
Very good	50.82
Good	38.36
Fair	8.59
Poor (ref.)	1.97
**Skipped meals**	
Rarely or never	71.92
All the time to sometimes (ref.)	28.08
**Self-rated health**	
Excellent	12.94
Very good	37.41
Good	34.10
Fair	13.19
Poor (ref.)	2.36
**Pain**	
None (ref.)	62.32
Mild	12.45
Moderate	20.04
Severe	5.19
**Resilience score**	42.14 (5.79)

**Table 2:** Linear regression for resilience score among older adults with multimorbidity (*N* = 13,064)

Variables	Beta
**Age**	-0.01
**Gender**	
Female	-0.01
Male (ref.)	
**Education level**	
Post-secondary education	0.02
No post-secondary education (ref.)	
**Household income**	
$20,000 to $49,999 per year	0.02
$50,000 to $99,999 per year	0.07**
$100,000 to $149,000 per year	0.08***
$150,000 and over per year	0.06***
Less than $20,000 per year (ref.)	
Not stated	0.02
**Marital status**	
Married/Common low	-0.03*
Not Married (ref.)	
**Immigration status**	
Born in Canada	0.03**
Immigrants (ref.)	
**Number of friends**	0.07***
**Number of relatives**	0.06***
**Housing problem**	
Yes	-0.06***
No (ref.)	
**Urban/Rural status**	
Urban area	-0.01
Rural area (ref.)	
**BMI**	
Underweight	0.02
Overweight	0.06***
Obese	0.07***
Normal (ref.)	
**Sedentary**	
Siting less than 1 h	-0.04***
1 h to less than 2 h	-0.01
2 h to less than 4 h	-0.01
4 h and more (ref.)	
**Alcohol consumption**	
14 drinks or less per week	0.03*
15 drinks or more per week (ref.)	
**Smoking**	
Not in the last 30 days	0.03**
Smoked (ref.)	
**Sleep**	
Very satisfied	0.19***
Satisfied	0.13***
Neutral	0.06***
Dissatisfied	0.05**
Very dissatisfied (ref.)	
**Appetite**	
Very good	0.16***
Good	0.11**
Fair	0.02
Poor (ref.)	
**Skipped meals**	
Rarely or never	0.04**
All the time to sometimes (ref.)	
**Self-rated health**	
Excellent	0.23***
Very good	0.22***
Good	0.14***
Fair	0.03
Poor (ref.)	
**Pain**	
Mild	-0.03**
Moderate	-0.02
Severe	0.01
None (ref.)	
**Adjusted R**^**2**^	0.112***

*p < .05

**p < .01

***p < .001

**Table 3:** Linear regression for resilience score among older adults with multimorbidity for three clusters of chronic health conditions

Variables	Cardiovascular/Metabolic cluster *n* = 3,828	Musculoskeletal cluster *n* = 3,691	Mental Health cluster *n* = 1,158
	Beta	Beta	Beta
**Age**	-0.02	-0.03	-0.09*
**Gender**			
Female	0.04*	-0.02	-0.04
Male (ref.)			
**Education level**			
Post-secondary education	0.01	0.02	-0.04
No post-secondary education (ref.)			
**Household income**			
$20,000 to $49,999 per year	0.04	-0.01	0.12
$50,000 to $99,999 per year	0.12**	0.05	0.15
$100,000 to $149,000 per year	0.07^*^	0.06	0.09
$150,000 and over per year	0.09**	0.04	0.12*
Less than $20,000 per year (ref.)	0.01	-0.01	-0.04
Not stated			
**Marital status**			
Married/Common low	-0.03	-0.05	-0.08
Not Married (ref.)			
**Immigration status**			
Born in Canada	0.01	0.01	0.01
Immigrants (ref.)			
**Number of friends**	0.05*	0.08*	0.03
**Number of relatives**	0.07***	0.08***	0.12**
**Housing problem**			
Yes	-0.09***	-0.08***	-0.06
No (ref.)			
**Urban/Rural status**			
Urban area	-0.02	-0.03	-0.05
Rural area (ref.)			
**BMI**			
Underweight	0.03	0.04*	0.04
Overweight	0.03	0.10***	0.10*
Obese	0.07^*^	0.09***	0.12^**^
Normal (ref.)			
**Sedentary**			
Siting less than 1 h	-0.06**	-0.06**	-0.02
1 h to less than 2 h	0.01	-0.01	-0.03
2 h to less than 4 h	0.01	-0.01	0.03
4 h and more (ref.)			
**Alcohol consumption**			
14 drinks or less per week	0.01	0.02	0.05
15 drinks or more per week (ref.)			
**Smoking**			
Not in the last 30 days	0.01	0.01	0.03
Smoked (ref.)			
**Sleep**			
Very satisfied	0.13***	0.16***	0.20**
Satisfied	0.07	0.14***	0.20**
Neutral	0.05	0.07*	0.09
Dissatisfied	0.02	0.07*	0.09
Very dissatisfied (ref.)			
**Appetite**			
Very good	0.15*	0.28^***^	0.27^*^
Good	0.07	0.20**	0.23*
Fair	-0.02	0.10*	0.01
Poor (ref.)			
**Skipped meals**			
Rarely or never	0.04*	0.04	0.02
All the time to sometimes (ref.)			
**Self-rated health**			
Excellent	0.14***	0.19***	0.07
Very good	0.16***	0.20***	-0.04
Good	0.08	0.12^*^	-0.11
Fair	-0.02	0.01	-0.07
Poor (ref.)			
**Pain**			
Mild	-0.04*	0.01	0.05
Moderate	-0.06**	0.01	0.01
Severe	0.01	0.05	0.05
None (ref.)			
**Adjusted R**^**2**^	0.131***	0.126^***^	0.193***

*p < .05

**p < .01

***p < .001

Finally, since the FUP2 data that included the CD-RISC_10 outcome measure were collected during the COVID-19 pandemic, we performed a supplementary analysis (not shown in the results). We re-ran all of the analyses controlling for a dichotomous variable measuring whether the CD-RISC_10 score was collected prior to March 16th, 2020 or from that date onward. None of the substantive findings were affected, and we only report the results without this control variable.

## Results

The mean age for participants is 73.75 (standard deviation (SD) = 6.7). Among all the participants, the majority were female (57%), highly educated (55%), receiving moderate level household income between $20,000 to $100,000 (66%), married (65%), born in Canada (79%), living in urban areas (96%), having no housing issue (80%). Participants reported an average of 5 close friends and 30 relatives. Roughly 3 out of 5 participants have more than 4 h sitting activities daily. Most of participants have 14 or less drink per week (93%) or did not smoke in the past 30 days before survey (95%). More than half of the participants were satisfied with their sleeping quality (61%), had good to very good appetite (69%), and never or rarely skipped meal (72%). About 70% of participants were overweigh (39%) or obese (31%). Almost 85 percent of participants rated their health as good to excellent, and most of them did not experience any pain (62%). The average resilience score for selected participants was 42.14 (SD = 5.79). More detailed information of participants characteristics is illustrated in Table 1.

Table 2 shows the results of the linear regression for resilience score among our sub-sample of multimorbid older adults. Standardized coefficients (β) can be interpreted as the change in the dependent variable (CD-RISC 10 scale, with a range of between 10 and 50) measured in standard deviation units for a one standard deviation change in each independent variable category, holding all other independent variables constant. This affords direct comparability of the strength of independent variables.

Household income is significantly related to resilience, where higher income groups (compared to less than $20,000) were associated with higher scores (β = 0.07** for $50,000 to $99,999 per year, β = 0.08*** for $100,000 to $149,000 per year, and β = 0.06*** for $150,000 and over per year). Married or partner participants reported a lower resilience scores than those without a spouse or partner (β = -0.03*). Participants born in Canada had a higher resilience score (β = 0.03**) than foreign-born. Both the number of close friend and relatives are positively related to resilience (β = 0.07*** and β = 0.06*** respectively). Participants with housing problems tend to report a lower resilience (β = -0.06***) than those without problems. Unexpectedly, participants who were overweight (β = 0.06***), or obese (β = 0.07***) reported higher scores than participants with normal weight. Also, participants with fewer sitting activities (less than 1 h of inactivity per day) had lower resilience than those with 4 h or more sitting activities (β = -0.04 *** for less than 1 h of siting activities). Participants who drank 14 drinks or less per week reported higher resilience scores than those with 15 or more drinks per week (β = 0.03*). In addition, the participants who did not smoke in the past 30 days (compared to those who did smoke) had slightly higher resilience scores (β = 0.03**). Compared to being very dissatisfied, participants with higher levels of satisfaction with sleeping quality all reported greater resilience (β = 0.19*** for very satisfied; β = 0.13*** for satisfied; β = 0.06*** for neutral; β = 0.05** for dissatisfied). Participants with very good (β = 0.16***) or good (β = 0.11**) appetite both had higher resilience scores than those rated their appetite as poor. Participants never or rarely skipped meals also reported higher resilience than those who skipped meals sometimes to all of the time (β = 0.04**). Participants who rated their health as good to excellent (β = 0.23*** for excellent, β = 0.22*** for very good, β = 0.14*** for good) also reported higher levels of resilience than those with poor self-rated health. Lastly, participants with mild pain reported slightly lower resilience scores than those without any pain (β = -0.03**).

The results for three chronic health condition clusters are listed in Table 3. The relationships between socio-demographic factors, social and environmental factors, behavioural and lifestyle factors, and the health context factors and resilience scores are quite similar to the analysis with all participants (older adults with multimorbidity). High income, more friends, more relatives, not having housing problems, being overweight or obese, greater satisfaction with sleeping, better appetite, and higher perceived health were associated with resilience across all multimorbidity clusters (see Table 3 for coefficients). Education level, immigration status, marital status, urban/rural status, alcohol consumption and smoking did not result in statistically significant relationships with resilience for the three multimorbidity clusters. A few differences across clusters were found. Age is inversely associated with resilience among those with a Mental Health cluster, but not for the Cardiovascular/Metabolic and Musculoskeletal clusters. In addition, less sedentary behaviour is weakly associated with resilience for the Cardiovascular/Metabolic and Musculoskeletal clusters, but not for mental health. Rarely or never skipping meals (compared to sometimes or always) is weakly associated with resilience only for the Cardio-Meta cluster only.

## Discussion

### Summary

Among older adults with multimorbidity, higher resilience scores were associated (in order of magnitude) with higher self-rated health, greater sleep satisfaction, better appetite, higher household income, more relatives and friends, being overweight (compared to normal weight), fewer housing problems, and fewer skipped meals. Weaker but statistically significant associations were found for non-smokers, less alcohol consumption, being more sedentary, less pain, non-married (compared to married), and among Canadian-born (compared to foreign). The statistically significant associations range from small (β = 0.03*) to moderate (β = 0.28***) for these analyses.

The analyses for the three multimorbidity clusters are similar to the analysis with the full sample of participants (older adults with multimorbidity). High income, more friends, more relatives, not having housing problems, being overweight or obese, greater satisfaction with sleeping, better appetite, and higher perceived health were associated with resilience across all multimorbidity clusters; however, resilience associations with education level, immigration status, marital status, urban/rural status, alcohol consumption and smoking were not supported. Variations across the clusters include: age is inversely associated with resilience among those with a Mental Health cluster, but not for the Cardiovascular/Metabolic and Musculoskeletal clusters. Being sedentary is associated with resilience for the Cardiovascular/Metabolic and Musculoskeletal clusters, but not for Mental Health. Finally, rarely or never skipping meals (compared to sometimes or always) is weakly associated with resilience only for the Cardiovascular/Metabolic cluster.

#### Contribution to Resilience Literature

We have witnessed a proliferation of resilience models applied to older adults, including applications to understanding the ability to adapt and recover from multimorbidity adversity [28, 43, 62, 70, 73, 93, 94, 96, 101, 103]. While many different measurements of multimorbidity resilience (MR) have been employed in this literature (see [71], no studies to date have used the Connor-Davidson [16] resilience scale for older persons with multiple concurrent chronic conditions. In addition, it is the most utilized measure in the broader resilience literature; it has been used in other multimorbidity research; and it includes several items related to bouncing back from disease/illness (see Measurement). The present study afforded an opportunity to compare findings associated with multimorbidity using this measure to confirm prior research using other measures of resilience. Indeed, our study is the first to examine predictors of resilience using the Connor-Davidson scale (CD-RISC 10) among older adults with multimorbidity, including sub-analyses using selected illness clusters.

The weak to moderate statistically significant associations between resilience among persons with multimorbidity and a spectrum of health behaviours and other SDoH, many of which entail modifiable risk factors, are consistent with a large body of literature in public health and health promotion (for example, [12, 20, 24, 53, 75, 77, 102]. Importantly, our study results parallel many of those uncovered for predictors of multimorbidity per se. For instance, Skivington et al.’s [77] Scottish longitudinal study of multimorbidity found support for a similar set of behavioural and lifestyle factors. Multimorbidity risk was higher among smokers compared to non-smokers,, for those with BMI 30–35, and > 35, compared to BMI 20–25; and for those with poor diet, after controlling for socio-demographic variables.

Turning to initial longitudinal research examining multimorbidity resilience among older adults, Wister et al. [100] developed and validated a multidimensional (functional, social and psychological) multimorbidity resilience index (MRI), and supported many of the same associations reported in the present study using the Connor-Davidson Resilience [16] CD-RISC 10 measure. These findings are important since both studies use the same CLSA data, a national Canadian longitudinal study, which thus provides confirmatory research on resilience among older persons with multimorbidity [71, 103]. The MRI used in prior research was shown to have good criterion validity (Wister et al. [100]), capturing multiple ways in which older adults can bounce back from adversity associated with having multiple concurrent chronic illnesses, although not used as extensively as the Connor-Davidson scale. The MRI was found to be associated with higher income, being married, fewer housing problems, more friends and relatives, higher perceived health, less pain and fewer medications [101, 103]. Among lifestyle behavioural factors, their index was associated with not smoking, greater sleep satisfaction, better appetite and not skipping meals. The similarity in predictors across the MRI resilience index measure and the Connor-Davidson resilience scale offers confirmatory evidence of several key modifiable lifestyle behavioural factors, as well as several indicators of socio-economic status, as well as housing problems.

Furthermore, our results are also consistent with a number of targeted studies on behavioural and lifestyle predictors of multimorbidity. Recently, researchers have begun to expand the more traditional list of health behaviours to include a wider range of lifestyle patterns, such as sleep quality and food security have received attention [72, 74, 77, 101]. Our findings provide supplementary knowledge for the salience of sleep quality in lowering health care utilization and enhancing illness treatment,in this case, as a positive health behaviour for illness coping and recovery in the face of multimorbidity, as well as role and identity reintegration [23, 69, 74]. We also found that having a good appetite and not skipping meals as forms of food security promote levels of resilience among those with multiple concurrent chronic illnesses, which coincides with prior research demonstrating the importance of food security for older adults more broadly [72, 77].

Additionally, being overweight or obese compared to normal weight (a marker for unhealthy lifestyles) negatively affects resilience among all multimorbid older adults, and among the three illness clusters. This aligns with prior research on the influence of obesity on multimorbidity [12, 77]. Its inverse association with resilience in our study implies that this health indicator may also be a risk factor for adapting to multimorbidity, and is consistent with findings associated with eating habits as found in earlier research (e.g., [3, 12, 101]). In addition, smoking status has been shown to be associated with multimorbidity risk in numerous studies (for example, [12, 20, 24, 53, 77]),and furthermore, appears to compromise resilience among older persons with multimorbidity due to its adverse effect on quality of life and psychological well-being associated with its addictive properties  [82]. The present study contributes only supports a weak effect of not smoking for resilience fortitude, and only for the full sample, not the three illness clusters. Our study also showed a weak positive association between being sedentary and resilience, but only for the full sample of all multimorbid persons. Whether this is reflective of the importance of rest for individuals experiencing complex health problems, greater stress among working individuals who may be more sedentary, or whether physical inactivity is a weak measure of a full range of physical activity levels and types require further research [9, 14, 19, 48]. Indeed, the inconsistent findings in the multimorbidity and aging literature for being sedentary and physical inactivity may be the result of differences in design, age of the target population, and measurement. And while a positive association between lower levels of drinking and resilience was found, the effect is very weak. These finding parallels equivocal results found in other studies of healthy aging and multimorbidity, including some cross-sectional studies showing that drinking protects against multimorbidity (e.g., [65]), which may be a causal directional issue, since some people quit drinking due to illness.

Another set of analyses were conducted on the associations of all health behaviours and covariates for the three multimorbidity clusters: Cardiovascular/Metabolic, Musculoskeletal and Mental Health. For the two physical multimorbidity clusters (Cardiovascular/metabolic, and Musculoskeletal), most of the findings reported for the full multimorbidity sample. The exception is the Mental-health cluster, where only sleep satisfaction was found to be associated with resilience. Another notable exception was that obesity was related to MRI in the Musculoskeletal cluster only, which may reflect the additional loading demands of obesity on the musculoskeletal system [90]. Furthermore, being underweight related to lower resilience in the cardiovascular/metabolic disease cluster only, both at baseline and over time. Underweight BMI has previously been associated with increased mortality in Canadian seniors [88]. It is possible that trajectories of body weight changes may have disease-specific impacts on health outcomes and resiliency. However, these results should be interpreted with caution. Finally, females reported higher resilience than males in the Cardiovascular/Metabolic cluster, but there were no significant sex differences in resilience in the Musculoskeletal and Mental Health clusters, suggesting more work in this area to extend our understanding of potential gendered aspects of coping with multimorbidity.

Several other SDoH also appear to be important in enhancing resilience, in particular, income level and an absence of housing problems. These determinants of health are consistent with studies on multimorbidity as well as early research on forms of resilience among older adults [12, 15, 47, 53, 70, 77, 96, 101]. Turning to the health context measures, higher multimorbidity resilience was associated with better perceived health and less perceived pain, although the latter association was very weak. Again, these findings concur with other research establishing that subjective illness dimensions that may affect perceptions of illness resilience [26, 41, 79, 91].

The findings from this study support several processes comprising the Lifecourse Model of Multimorbidity Resilience (LMMR). The model contends that the ability to bounce back from multimorbidity adversity is influenced by the nexus of individual, social and environmental-level resources or deficits. The salience of several health behaviours, socio-economic status, and housing problems supports the model. Social support factors were less important. Future research needs to explicate the ways in which these domains interact, as well as life course trajectories affecting resilience among persons with multimorbidity.

### Limitations

Several limitations of these analyses are notable. First, we employed a cross-sectional design given that the outcome variable was only available in the FUP2 wave. Future waves of the CLSA will allow employing longitudinal analyses of the CD-RISC 10 resilience measure. Second, since multimorbidity is variable due to differing symptom presentation and illness severity (e.g., hypertension, cancer, diabetes, etc.), research that incorporates additional illness context factors, such as onset, severity, and duration, is needed [71]. The lack of severity measurement could have contributed to the weak-moderate effects identified in this research. There are also other independent variables not included in this study that could improve the specification of the model and its overall explanatory ability. Since we did not include severity, we combined persons with significant variations in multimorbidity, which can dilute some of the findings. Third, chronic illnesses are self-reported and may be influenced by individual memory and health literacy. Fourth, similar to multimorbidity, there may be clusters of health behaviours that generate cumulative effects of modifiable health behaviours over time, which has been useful in the broader multimorbidity risk literature [20, 24, 80]. This could include combinations of not smoking, sedentary behaviour, maintaining positive eating habits and a healthy weight, and quality sleep, as well as variations in risk levels for each condition [80], which may in conjunction help to specify the treatable, or modifiable moments in illness trajectories to develop more effective public health strategies. It should also be noted that obesity is a marker of other health behaviours, such as eating habits and sedentary behaviour or physical inactivity. Fifth, this work needs to be extended to a variety of diverse groups that may have unique behavioural and illness contexts, such as racial/ethnic groups [58, 71], those without health care insurance [15, 86], as well as during other forms of adversity such as the COVID-19 pandemic [39, 43, 63, 104]. Finally, other analytic approaches are worthy of attention, in particular, incorporating non-multimorbid individuals for comparisons, longitudinal analyses, and using multiplicative interaction terms for the cluster-risk factor analysis.

## Conclusion

The ability to adapt, bounce back or reintegrate from multiple chronic illnesses, termed multimorbidity resilience, is fundamental to healthy aging and is receiving increasing attention in the literature [15, 46, 57, 62, 71, 73, 101], including pandemic research [63, 104]. Our findings indicate that there are several mutable health behaviours that are associated with resilience among older persons with multimorbidity worthy of considering for intervention development and public policy. The health behaviours found to be important in this study can be used to tailor and target health promotion and public health programs and policies. In particular, some of these have not received the attention that they deserve, such as sleep quality. New public health initiatives could also investigate strategies to improve multiple health behaviours that affect persons with multimorbidity, rather than single ones; however, more work is needed in this area. Innovations in the delivery of interventions for older adults with multimorbidity may utilize a multipronged set of health promotion approaches (e.g., multifactor telehealth counselling, digital behavioural monitoring devices, community support programs, peer support groups, tailored cognitive therapy, etc.). Indeed, proactive, strength-based approaches to enhance resilience may prove to be valuable in enhancing perceptions of resilience and more positive health outcomes. Finally, several known social determinants of multimorbidity also have been found to be important, including age, gender, socio-economic status/deprivation factors and social support, which may also be low hanging fruit in the development of interventions targeting resilience. The present study serves to advance important findings for other studies to build upon regarding the complex ways in which resilience can be elucidated and enhanced among persons experiencing multimorbidity over the life course.

## Data Availability

The CLSA data are available at (https://www.clsa-elcv.ca/).

## Abbreviations

MR: Multimorbidity resilience
LMMR: Lifecourse Model of Multimorbidity Resilience
SDoH: Social determinants of health
CLSA: Canadian Longitudinal Study on Aging
CD-RISC 10: Connor-Davidson Resilience Scale 10 item scale
